# Prevalence of Vision Problems and Associated Risk Factors Among Secondary Government School Girls: A Descriptive Cross-Sectional Study in Western Maharashtra, India

**DOI:** 10.7759/cureus.87281

**Published:** 2025-07-04

**Authors:** Pooja S Sohil, Sudhanshu A Mahajan, Sumeet M Vaidya, Rupeshkumar B Deshmukh, Saibal Adhya

**Affiliations:** 1 Community Medicine, Bharati Vidyapeeth Deemed to be University Medical College, Pune, IND

**Keywords:** government schools, prevalence, pune, risk factors, vision problems, visual acuity

## Abstract

Background: Visual health is crucial for academic and psychosocial development in school children. The increasing use of digital devices and reduced outdoor activities post-COVID-19 have contributed to rising vision problems, especially in school children. However, there is still limited data available from urban government schools in India.

Methods: A community-based cross-sectional study was conducted among 420 girls from 8th and 9th grades in a randomly selected government girls' school under the Urban Health Training Centre in Pune, Western Maharashtra. Vision screening was performed using Snellen charts, and socio-demographic data were collected through a validated, pretested questionnaire. Data collection included face-to-face interviews and vision problems checked by ophthalmology residents. Associations between vision problems and risk factors were assessed using chi-square tests and odds ratios; a p-value of <0.05 was considered statistically significant.

Results: The prevalence of abnormal visual acuity overall was 168 (40%), with 67 (16%) mild, 92 (22%) moderate, and 9 (2%) severe. Key risk factors included frequent eye rubbing, excessive blinking, and inadequate lighting at home, all significantly associated with poor visual acuity (p<0.05). Among students with vision acuity, 53 (31.5%) were reluctant to wear spectacles. Additionally, awareness among parents, affordability, and accessibility also emerged as notable barriers to the utilization of eye health services.

Conclusion: A structured school-based eye health program is crucial for early detection, educating parents, and ensuring equal access to vision care, thereby reducing preventable visual acuity issues among school girls in India.

## Introduction

Worldwide, over 7 out of every 100 children are visually impaired due to uncorrected visual acuity [1]. The reported prevalence of blindness in low- and middle-income countries varies from 0.2 to 7.8 cases per 10,000 people. In developed and industrialized countries, the annual incidence of blindness is six cases per 10,000 children under the age of 15 [2]. Vision plays a critical role in children's cognitive, social, and physical development. Clear and healthy vision is essential for academic achievement, as it directly influences learning abilities, classroom performance, and involvement in extracurricular activities [3]. Since the outbreak of the novel coronavirus in December 2019, India imposed a nationwide lockdown starting in March 2020 [4]. Like children in other countries, school children in India were confined to their homes and transitioned to online education. The reduction in outdoor activities and the increase in screen time have been linked to the exponential rise in visual impairments among children [5]. The increasing use of digital devices among school-aged children has raised concerns about digital eye strain and other vision-related issues like refractive errors at a very young age [6].

India was the first country in the world to launch the National Program for Control of Blindness in 1976, aiming to reduce the prevalence of blindness to 0.3% by the year 2020. In 1999, the WHO launched Vision 2020: Right to Sight to eliminate avoidable blindness from refractive errors, cataracts, xerophthalmia, trachoma, and other causes of childhood blindness by the end of 2020 [7]. In 2013, the World Health Assembly adopted the "Universal Eye Health: Global Action Plan 2014-2019," which aims to reduce the prevalence of avoidable visual impairment by 25% by the year 2019, relative to the baseline prevalence recorded in 2010. In response to this initiative, India has undertaken a series of measures within its ongoing National Program for Control of Blindness and Visual Impairment to address issues related to blindness and visual impairment effectively [8].

Refractive errors, particularly myopia (near-sightedness), have become increasingly prevalent on a global scale [9]. Myopia represents one of the most common vision impairments and currently affects over 2.2 billion individuals worldwide. Research indicates that by the year 2050, it is anticipated that 50% of the global population may be affected by myopia [10]. It is important to note that many school-aged children are often unaware of their visual deficiencies and typically do not voice concerns regarding their eyesight. Frequently, they compensate for their visual challenges by altering their seating arrangements in the classroom, moving closer to objects, or intentionally avoiding activities that require greater visual concentration. Children's visual condition is influenced by environmental, lifestyle, and hereditary factors [11]. Even minor refractive issues can lead to headaches, concentration difficulties, and impaired coordination, resulting in academic challenges. These conditions may be asymptomatic, leaving children unaware of their vision problems, which may therefore go unrecognized by parents and educators. Early detection and correction of vision problems can provide significant educational and behavioral benefits, ultimately enhancing quality of life.

Despite the presence of school-based eye health programs aimed at early detection of vision problems, data on the prevalence and associated risk factors among secondary school girls in Western Maharashtra in Pune city are limited. Identifying these issues early can help implement effective interventions, reduce the burden of undiagnosed vision problems, and enhance the overall well-being and academic success of girls. The present study seeks to address this knowledge gap by achieving the objectives such as estimating the prevalence of vision problems among secondary school girls in Pune, determining the associated risk factors of vision problems, and exploring the reasons for non-utilization of eye health services.

## Materials and methods

Study design, population, and sampling

A cross-sectional, community-based study was conducted among secondary school girls enrolled in government schools associated with the Urban Health Training Centre (UHTC) of a private medical college located in Pune, Western Maharashtra, India. The UHTC provides health-related services to three government schools; from these, one girls' school was randomly selected for the study. The targeted population included girls in the eighth and ninth grades who were present during the data collection period and provided assent, alongside written informed consent from their parents or guardians. Students with known congenital eye disorders or systemic illnesses that may affect their vision were excluded from participation in the study.

Ethical consideration

Before the study, approval was granted by the institutional ethics committee (BVDUMC/IEC/75/25-26). Students were informed about the study's objectives, and their concerns were addressed. No personal identifiers were included in the datasets, and investigators reviewed all forms for completeness and accuracy.

Sample size calculation

The sample size for this study was calculated based on a reported prevalence of refractive errors of 22.14%, derived from a study conducted in urban India [12]. Using a 95% confidence level, an allowable error of d = 4.43 (20% of prevalence) was established, leading to a minimum required sample size of 338 participants. In total, 420 girls in the 8th and 9th grades were assessed for vision-related issues.

Data collection

The study was carried out over six months from February 2025 to April 2025. A health check-up camp was organized for girls in the 8th and 9th grades at the selected school, following the principal's approval. We explained the study's purpose, methods, and potential benefits to gain their support. The camp was scheduled on a predetermined date established by the school management. Eight days prior to the planned health check-up camp, the investigator, accompanied by a trained social worker, visited the respective classes to distribute consent forms and socio-demographic information forms to the students. Both forms were subsequently collected during the health check-up camp.

Data collection tool

A pre-designed, semi-structured, validated, and pre-tested questionnaire was used for data collection which was translated into the local language. The questionnaire was developed after a thorough review of the literature and included sections on socio-demographic profiles of parents, student information, vision problems, risk factor assessment of both eyes, and vision screening using the Snellen chart. The questionnaire was initially prepared in English, then translated into Marathi, and subsequently back-translated into English to ensure the accuracy of the translations. A pilot study was conducted with 38 school students in a similar setting, and necessary modifications were made based on the findings of the pilot study. Data was collected with the help of Google Forms by a principal investigator along with a trained medical social worker using a face-to-face interview technique, which lasted approximately 20 minutes per respondent. The vision screening was done by ophthalmology residents.

Operational definitions

Vision Problems

Any condition that affects normal visual function, including refractive errors (myopia, hyperopia, astigmatism), strabismus, amblyopia, and other diagnosed ocular issues. 

Visual Acuity

The sharpness or clarity of vision is measured using a Snellen chart [13], recorded as the smallest line of letters a person can read from a standard distance (6 meters). Severity of Visual Acuity (WHO Classification) [14]: Mild: Visual acuity <6/9 to 6/12, Moderate: <6/18 to 6/60, Severe: <6/60 to 3/60, Blindness: <3/60.

Statistical analysis

All statistical analyses were performed using IBM SPSS Statistics for Windows, Version 29 (Released 2023; IBM Corp., Armonk, New York, United States). Quantitative variables were presented as descriptive statistics (mean, SD), while qualitative variables were summarized as frequencies and percentages (%). The Chi-square test was used to assess associations between demographic variables, different risk factors, and visual acuity of the worst eye of school girls. Binary logistic regression was performed to determine the strength of association and estimate odds ratios (ORs) for visual acuity of the worst eye of school girls across socio-demographic variables and different risk factors. Results were reported with 95% confidence intervals (CIs), and a p-value < 0.05 was considered statistically significant.

## Results

Table 1 assessed the sociodemographic characteristics of 420 secondary government school girls. A majority of the students, 265 (63.1%), were from the 9th grade, and over half (221; 52.6%) belonged to the open caste. A maximum (302; 71.9%) of the students came from nuclear families, which aligns with the cultural norms observed in metropolitan areas. Regarding parental education, 227 (54%) fathers and 192 (45.7%) mothers had education levels above 10th grade. Skilled occupations were more common among fathers (334; 79.5%), whereas a higher proportion of mothers (272; 64.8%) were engaged in unskilled work. Socioeconomic status (SES) was predominantly higher, with 385 (91.7%) students belonging to SES I, which included upper class and upper middle class. Environmental conditions for the study were largely favorable, as 412 (98.09%) reported adequate lighting at home. Additionally, 279 (66.4%) children had screen time limited to one hour or less. These findings suggest that most students had a relatively supportive home environment, though disparities in maternal education and occupation highlight areas for potential intervention.

**Table 1:** Socio-demographic characteristics of secondary school girls (n=420) *Unskilled worker includes a homemaker and the unemployed

Socio-demographic variables	Variables	Frequency (n)	Percentage (%)
Grade	8^th^	155	36.9
	9^th^	265	63.1
Religion	Hindu	413	98.3
	Muslim	7	1.7
Caste	Open	221	52.6
	Others	199	47.4
Type of family	Joint	118	28.1
	Nuclear	302	71.9
Education of father	>10^th^ grade	227	54.0
	≤10^th^ grade	193	46.0
Education of mother	<10^th^ grade	228	54.3
	>10^th^ grade	192	45.7
Occupation of mother	Skilled worker	148	35.2
	*Unskilled worker	272	64.8
Occupation of father	Skilled worker	334	79.5
	*Unskilled worker	86	20.5
Socioeconomic status (SES)	I (Upper Class) + II (Upper Middle Class)	385	91.7
	III (Middle Class) + IV (Lower Middle Class)	35	8.3
Lighting at home	Adequate	412	98.09
	Inadequate	08	1.9
Screen timing	<1 hour	279	66.4
	>1 hour	141	33.6

**Figure 1:**
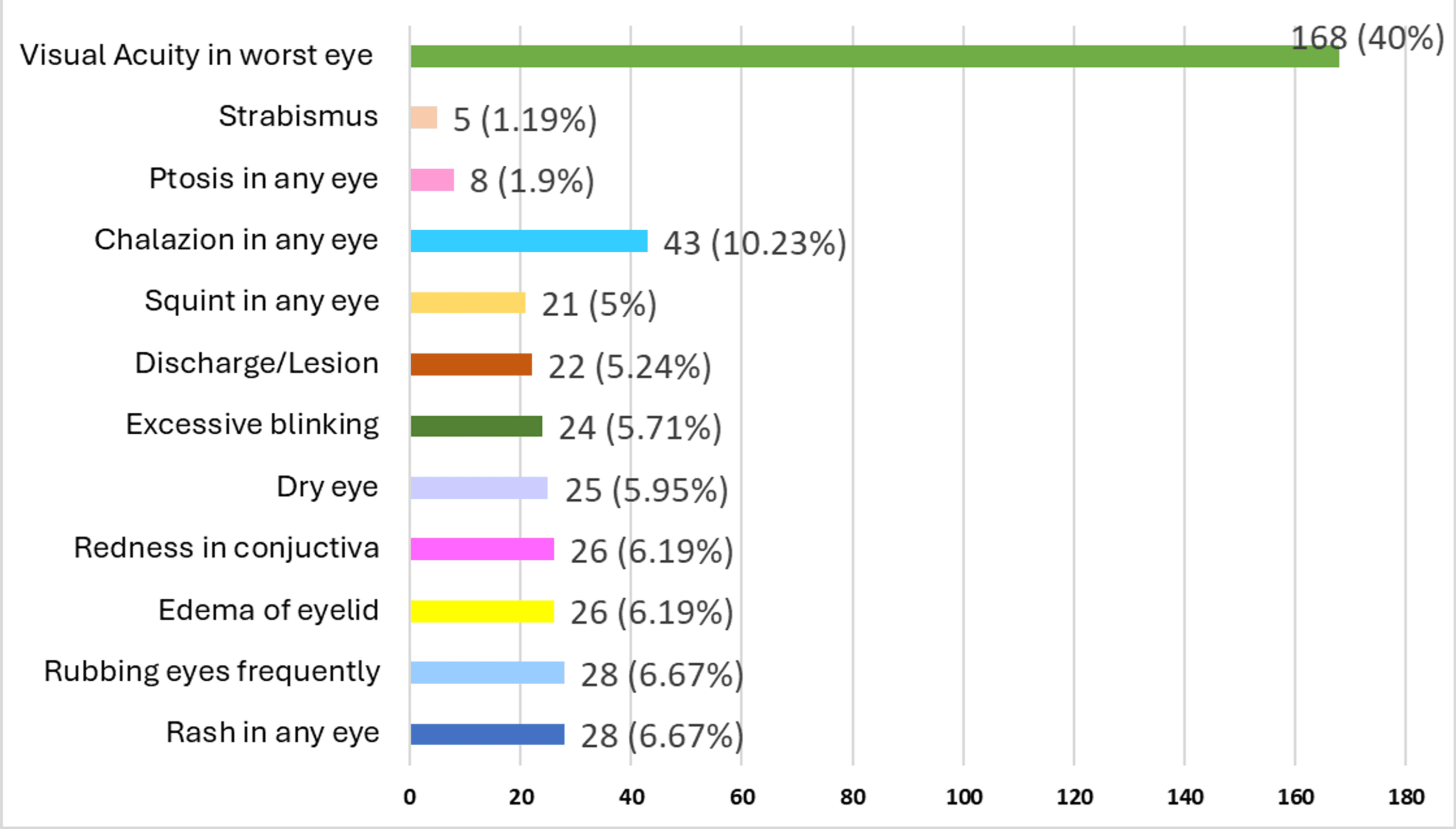
Prevalence of vision problems in secondary school girls (n=420)

Figure 1 depicts the prevalence of various vision problems among 420 secondary school girls. The most commonly reported issues were rash and eye rubbing, each affecting 28 (6.67%) of the girls. This was followed closely by redness in 26 girls (6.19%), edema in 26 girls (6.19%), and dry eye in 25 girls (5.95%). Other notable complaints included excessive blinking in 24 girls (5.71%), discharge or lesion in 22 girls (5.24%), and squint in 21 girls (5%). Less common problems included ptosis in eight girls (1.90%), chalazion in 43 girls (10.23%), and strabismus in five girls (1.19%). The data also reveals that 168 (40%) of the school girls exhibited varying degrees of visual acuity.

**Figure 2:**
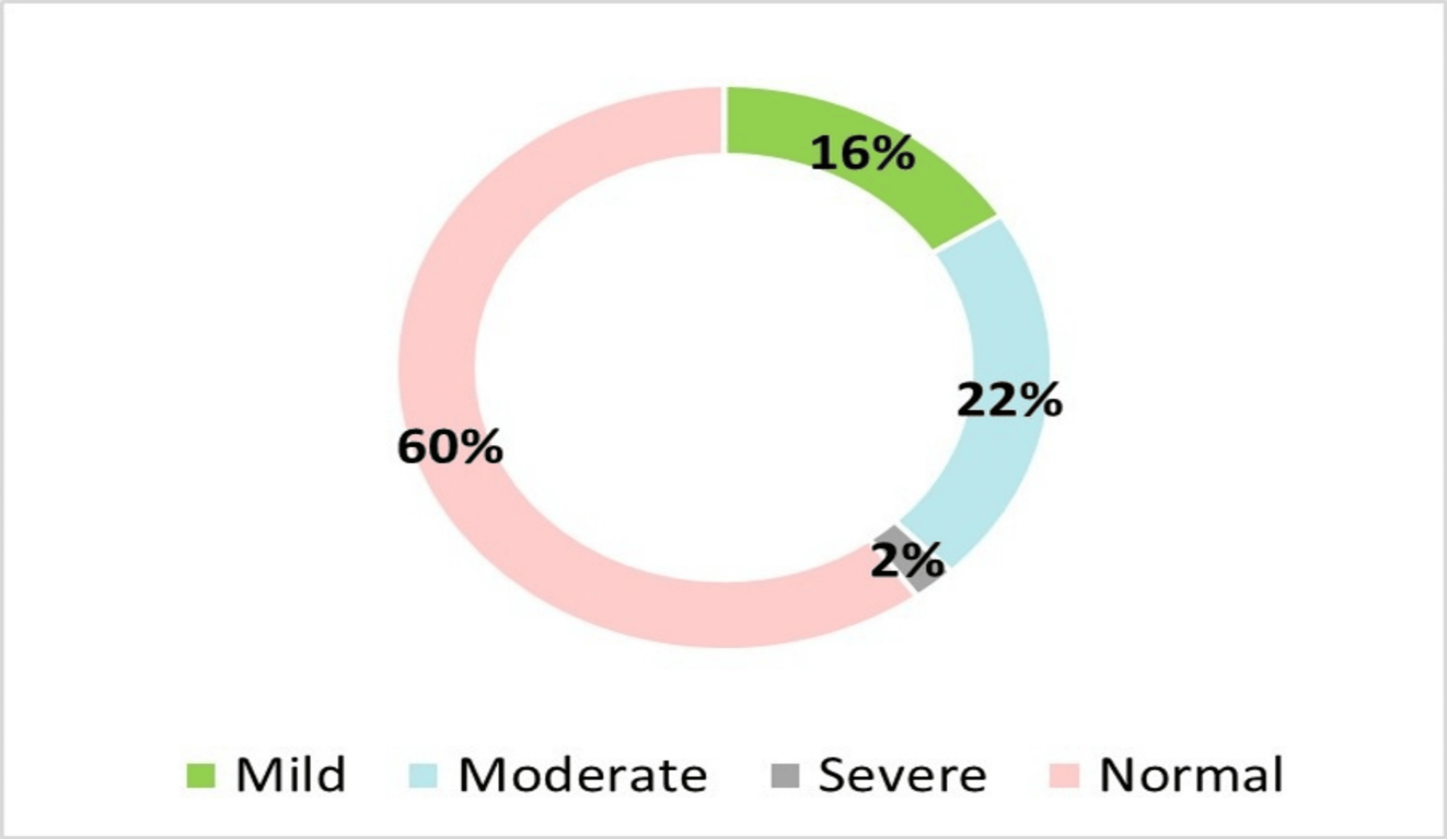
Prevalence of visual acuity of the worst eye in secondary school girls (n=420)

Figure 2 illustrates a pie chart of the prevalence of visual acuity in the worst eye among 420 secondary school girls. The data indicate that 252 (60%) of the students had normal vision according to World Health Organization (WHO) criteria, while 168 (40%) exhibited varying degrees of visual acuity. Of these, 67 (16%) had mild, 92 (22%) had moderate, and 9 (2%) had severe visual acuity. These findings underscore the need for routine vision screenings and early corrective measures in school health programs.

**Figure 3:**
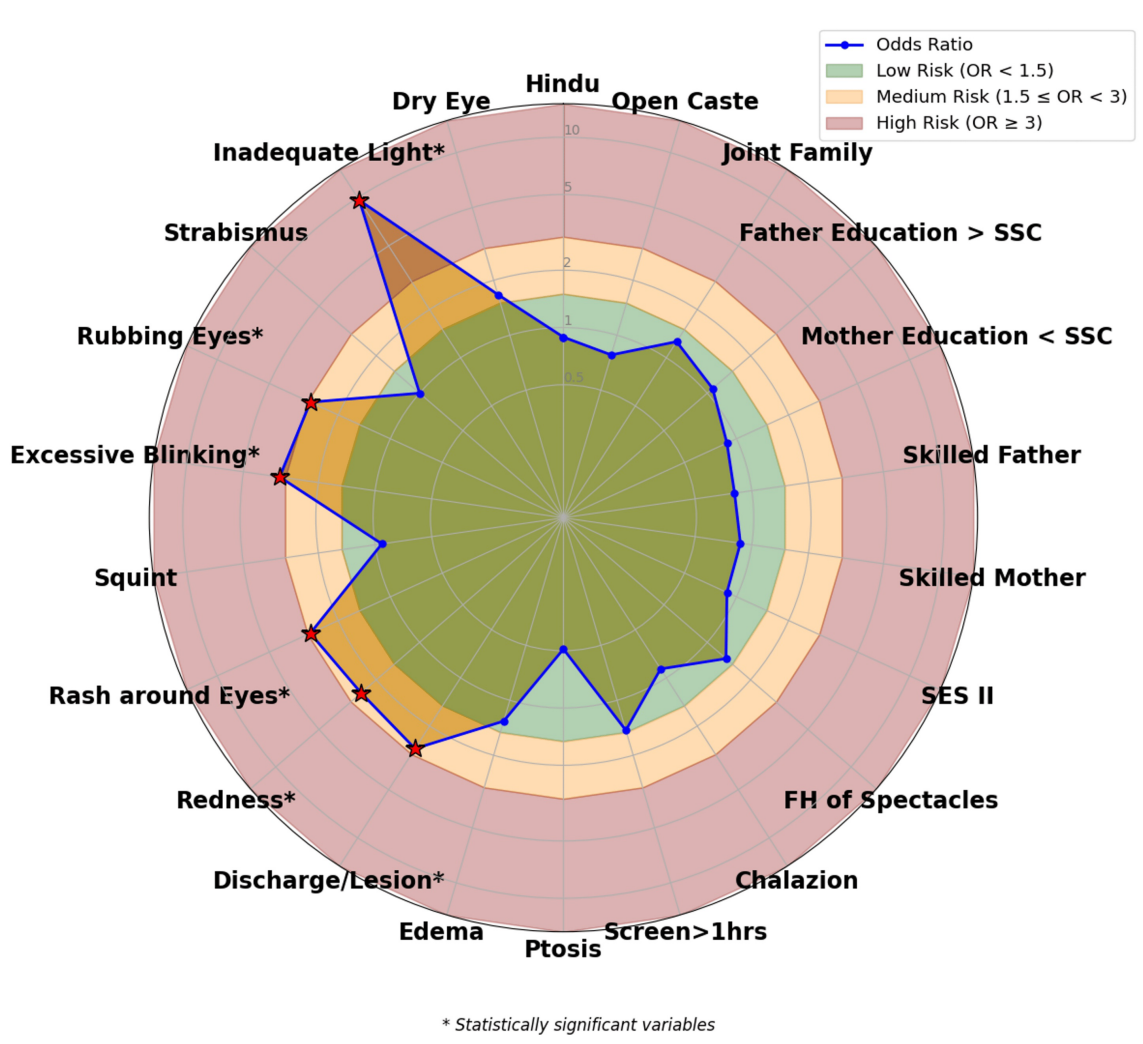
Strength of association between risk factors and visual acuity in secondary school girls (n=420)

Figure 3 presents a radar graph depicting various risk factors associated with visual acuity among 420 school children. The chart categorizes these factors into low (green zone), medium (yellow zone), and high-risk (red zone). Notably, statistically significant risk factors are marked with a red asterisk, which include excessive blinking, rash around the eyes, redness in the conjunctiva in any of the eyes, rubbing the eyes frequently, and inadequate lighting at home, indicating a strong association with visual acuity of the worst eye.

Table 2 represents the association of socio-demographic variables with visual acuity of the worst eye. The association was analyzed by the chi-square test and odds ratio. Among all the socio-demographic variables studied, only adequate lighting at home demonstrated a statistically significant association with visual acuity. Participants exposed to inadequate lighting at home had significantly higher odds of having vision impairment than those exposed to adequate lighting at home. (OR = 9.61, 95% CI: 2.12-43.54; p = 0.003). The corresponding chi-square test also showed a statistically significant difference (χ² = 12.61, p < 0.001), underscoring the robustness of this association. This finding highlights the potential role of environmental factors-specifically, poor illumination-in the development or exacerbation of visual impairments. Other socio-demographic variables such as grade level, religion, caste, type of family, parental education and occupation, socioeconomic status, and a family history of spectacles did not show statistically significant association with visual acuity of the worst eye (p-values > 0.05).

**Table 2:** Association of socio-demographic variables with visual acuity of the worst eye by the chi-square test and odds ratio (n=420) * Fisher's exact test The values in bold are statistically significant (p < 0.05)

Socio-demographic variables		Visual acuity of the worst eye		Total	Chi-square value	p-value	OR (95%CI)	p-value
		Vision impairment (n=168)	Normal (n=252)					
Grade	8th	58	97	155	0.68	0.41	0.84(0.56-1.26)	0.41
	9th	110	155	265			1	
Religion	Hindu	165	248	413	Fisher's exact test	0.99*	0.89(0.20-4.01)	0.88
	Muslim	3	4	7			1	
Caste	Open	82	139	221	1.63	0.21	0.78(0.52-1.14)	0.20
	Others	86	113	199			1	
Type of family	Joint	52	66	118	1.13	0.29	1.26(0.82-1.94)	0.29
	Nuclear	116	186	302			1	
Education of Father	>SSC	93	134	227	0.19	0.66	1.09(0.74-1.62)	0.66
	≤SSC	75	118	193			1	
Education of Mother	≤SSC	88	140	228	0.41	0.52	0.88(0.59-1.30)	0.52
	>SSC	80	112	192			1	
Occupation of Father	Skilled	130	204	334	0.79	0.37	0.81(0.49-1.30)	0.37
	Unskilled	38	48	86			1	
Occupation of Mother	Skilled	56	92	148	0.51	0.51	0.87(0.58-1.31)	0.50
	Unskilled	112	160	272			1	
Socioeconomic status	I +II	153	232	385	0.13	0.72	0.88(0.44-1.77)	0.72
	III +IV	15	20	35			1	
Family history of Spectacles	Yes	81	103	184	2.21	0.14	1.35(0.91-2.00)	0.14
	No	87	149	236			1	
Lighting at home	Inadequate	6	2	08	12.61	<0.001	9.61(2.12-43.54)	0.003
	Adequate	162	250	412			1	
Screen timing	>1	65	76	141	3.29	0.07	1.46(0.97-2.20)	0.07
	<1	103	176	279			1	

Table 3 describes the association of vision problems with the visual acuity of the worst eye by the chi-square test and odds ratio. Several vision problems were found to be significantly associated with vision impairment of visual acuity in the worst eye. Secondary school girls presenting with discharge or lesions in the eye (OR = 2.77, 95% CI: 1.13-6.76, p = 0.03), redness of the conjunctiva (OR = 2.55, 95% CI: 1.13-5.76, p = 0.02), rash around the eyes (OR = 2.90, 95% CI: 1.30-6.46, p = 0.009), excessive blinking (OR = 3.21, 95% CI: 1.34-7.68, p = 0.008), and frequent eye rubbing (OR = 2.90, 95% CI: 1.31-6.45, p = 0.009) had significantly higher odds of vision impairment of visual acuity as compared to those without these vision problems. These associations suggest that such vision problems may serve as early clinical indicators of visual impairment, warranting prompt evaluation and intervention, especially in school-based child eye health screening programs.

**Table 3:** Association of vision problems with visual acuity of the worst eye by the chi-square test and odds ratio (n=420) *Fisher's exact test The values in bold are statistically significant (p < 0.05)

Vision problems		Visual acuity of the worst eye		Total	Chi-square value	p-value	Odds ratio (95%CI)	p-value
		Vision impairment (n=168)	Normal (n=252)					
Ptosis in any of the eyes	Yes	2	6	8	Fisher's exact test	0.49*	0.49(0.10-2.48)	0.39
	No	166	246	412			1	
Edema of the eyelid in any of the eyes	Yes	12	14	26	0.43	0.51	1.30(0.58-2.88)	0.52
	No	156	238	394			1	
Discharge/lesion in any of the eyes	Yes	14	8	22	5.4	0.02	2.77(1.13-6.76)	0.03
	No	154	244	398			1	
Redness in conjunctiva in any of the eyes	Yes	16	10	26	5.36	0.02	2.55(1.13-5.76)	0.02
	No	152	242	394			1	
Rash around the eyes	Yes	18	10	28	7.37	0.02	2.90(1.30-6.46)	0.009
	No	150	242	392			1	
Squint in any of the eyes	Yes	8	13	21	0.03	0.86	0.92(0.37-2.27)	0.86
	No	160	239	399			1	
Excessive blinking	Yes	16	8	24	7.54	0.006	3.21(1.34-7.68)	0.008
	No	152	244	396			1	
Rubbing the eyes frequently	Yes	18	10	28	7.37	0.007	2.90(1.31-6.45)	0.009
	No	150	242	392			1	
Strabismus	Yes	2	3	5	Fisher's exact Test	0.99*	1.00(0.17-6.05)	0.99
	No	166	249	415			1	
Chalazion	Yes	16	27	43	0.16	0.69	0.88(0.46-1.68)	0.69
	No	152	225	377			1	
Dry Eye	Yes	13	12	25	1.6	0.21	1.67(0.74-3.77)	0.21
	No	155	240	395			1	

Table 4 highlights the reasons cited by 168 students with vision impairment of visual acuity for not utilizing eye health services. The most common reason, reported by 53 (31.5%) of students, was reluctance to wear spectacles. Additionally, 35 (20.8%) mentioned that no parent or guardian was available to accompany them, indicating logistical challenges. About 25 (14.8%) students believed their vision was normal, reflecting a lack of awareness. Financial constraints were another barrier, with 20 (11.9%) stating they couldn't afford an eye check-up. Other reasons included headaches after wearing spectacles (16; 9.5%) and the distant location of health facilities (15; 8.9%), and a small proportion (4; 2.3%) reported that their parents or guardians did not believe in eye health checkups. These findings underscore the need for targeted interventions to address both perceptual and practical barriers to eye care utilization among school children.

**Table 4:** Reasons for non-utilization of eye health services in secondary school girls (n=168)

Reasons for non-utilization of eye health services (n=168)	Frequency (%)
Don’t want to wear spectacles	53(31.5)
No parent/guardian is free	35(20.8)
Students perceived that vision was normal for them	25(14.8)
No money for eye health check-up	20(11.9)
Headache after wearing spectacles	16 (9.5)
Health facilities are far	15 (8.9)
Parents/guardians don’t believe in eye health checkups	4 (2.3)

## Discussion

Prevalence of visual acuity

The prevalence of visual acuity observed in the present study is notably higher (168; 40%), which underscores the importance of early identification and correction of visual acuity in school-aged children. Banerjee et al. [11] conducted a study on school-aged children (6-12 years) from urban and rural districts of western Maharashtra, Pune and found that the prevalence of myopia was higher in urban areas (109; 94.8%) compared to rural areas (106; 70.7%). Their study reported a greater prevalence of visual acuity issues in urban areas than that observed in our study (168; 40%), which could be attributed to their larger sample size. In a study conducted by Mohandas et al. [15] among adolescents in the southern part of India, 406 (4.1%) had impaired vision, while our findings showed a prevalence rate of 9 (2%) among urban adolescent girls. Prabhu et al. [16] conducted a study in both urban and rural settings in the Udupi district of Karnataka, India, which found that the prevalence of visual impairment was 4.32%. This figure is significantly different from our findings. This discrepancy may be because the study was not conducted in government schools. Srivastava et al. [12] stated that refractive error is a significant cause of visual impairment globally. In their multistate study in India, Joseph et al. [17] suggested that refractive errors, particularly myopia, are very common in the country. They found that differences in prevalence between states may be influenced by literacy rates, indicating that the burden of myopia could increase as literacy improves.

Prevalence of vision problems

Narayanan et al. [10] conducted a study in a tribal school in South India, noting that unilateral vision impairment was observed in five (0.13%) children, all of whom presented with strabismus. This finding aligns with our study, in which five (1.19%) girls exhibited strabismus. Vidya and Kiran [18] conducted a study on higher primary school children in rural areas of Mangalore and found a ptosis prevalence of 8 (8.2%), which is higher than our prevalence of 8 (1.90%). This difference may be attributed to the study population being from rural areas. Pukrushpan et al. [19] conducted a case-control study examining excessive blinking in children, which revealed that four participants (4%) had dry eyes. This finding is somewhat consistent with our study, in which the prevalence of dry eyes was reported among 25 (5.95%) girls.

The radar chart of risk factors for visual acuity demonstrated that several modifiable and non-modifiable factors contributed to visual health. These included excessive screen time (>1 hour), inadequate lighting at home, and the presence of ocular symptoms such as redness of eyes, squint, and excessive blinking. Social determinants like low socio-economic status, lack of parental education, and unskilled parents also played a role. Children with a family history of spectacle use were more likely to experience visual acuity, indicating a genetic predisposition. These findings emphasize the multifactorial nature of visual impairment and the need for comprehensive school-based vision screening programs integrated with community awareness and parental education. A systematic review of the effects of light on children indicates that increased exposure to daylight is generally linked to improvements in ophthalmological, psychological, and physical health outcomes [20]. Additionally, a cluster-randomized controlled trial conducted in Taiwan found that exposure to outdoor light is associated with less progression of myopia, reduced axial elongation, and a 54% lower risk of myopia advancement [21]. Frequent eye rubbing among schoolchildren can indicate visual problems and may be associated with serious conditions such as keratoconus [22]. Our study reveals a significant correlation between the frequency of eye rubbing and the presence of visual acuity issues. Additionally, Hatt et al. [23] reported that 35 (29%) children experienced diplopia, with eye rubbing being the predominant symptom reported by 29 (83%) of those children. This underscores the importance of monitoring eye-related behaviors in children for early identification and intervention.

Reasons for non-utilization of eye health services

The present study reveals that the majority of school-going girls, 53 (31%), do not want to wear spectacles, despite having visual impairments. Khatri et al. [24] conducted a mixed-method study on adolescents in community schools and, through thematic analysis, highlighted the influence of aesthetic appearance, affordability, and accessibility on this issue. Hussain et al. [25] conducted a study in rural Bangladesh on barriers to accessing eye health services for children. They found significant obstacles, including a lack of knowledge, awareness, and financial constraints that hinder families from seeking proper healthcare. Our study's findings align with these observations.

India had successfully integrated primary eye health care for children into the Ayushman Bharat - Health and Wellness Centres [26], Rashtriya Bal Swasthya Karyakram [27], Universal Eye Health Coverage (under WHO Vision 2020) [28], Mobile Ophthalmic Units and District Blindness Control Societies where identifying eye problems and strong referral mechanisms at district hospitals were developed. Community health workers attached to the facilities such as Accredited Social Health Activists, Auxiliary Nurse Midwives, and Primary Health Care doctors in basic eye care were also engaged to promote awareness about eye conditions in children in the community.

Recommendations

The findings of the study will contribute to the formulation of effective policies aimed at enhancing the health-seeking behavior of parents. This includes removing existing barriers to the utilization of eye health services, strengthening the training of primary care providers, expanding tele-ophthalmology, and increasing public awareness through Information, Education, and Communication (IEC) activities. Additionally, it emphasizes better integration of eye care services into routine primary health care.

Strengths and limitations

This study is the first community-based research focused on a government school for girls in urban areas of western Maharashtra, specifically Pune. It aimed to identify the prevalence and risk factors of vision problems, as well as the reasons for the underutilization of eye health services. Policymakers can use these findings to develop an action plan aimed at reducing visual acuity issues by promoting coordination within and between sectors. However, it is important to note that the study was conducted in only one government urban girls' school, meaning it does not represent the entire nation.

## Conclusions

This study underscores the crucial importance of structured eye health programs in schools as a vital public health priority. The high incidence of undetected refractive errors among schoolchildren calls for regular vision screenings in educational settings. Providing affordable, quality spectacles and increasing awareness about eye health can aid in the early detection and management of visual impairments. Integrating these programs into school health systems can enhance children's academic performance and quality of life while reducing the long-term burden of preventable visual disabilities. Effective coordination among the health, education and social welfare department is crucial for the success of these initiatives. A comprehensive school eye health strategy is key to promoting school health and educational equity.

## Appendices

Pre-tested Questionnaire

(Eye health screening of school children)

1. Registration number of the participant:

2. Age (in yrs):

3. Gender: 

Male

Female

4. Class:

5. Use of spectacles:

Yes

No

6. Religion:

Hindu

Muslim

Christian

Buddha

Others

7. Caste:

General/open

OBC

ST/SC

Others

8. Type of Family:

Nuclear

Joint

Others

9. No. of Family Members:

10. Education of the Father:

Illiterate

Read and write

Less than 7

SSC

HSC

Graduate

Postgraduate

11. Education of the Mother:

Illiterate

Read and write

Less than 7

SSC

HSC

Graduate

Postgraduate

12. Occupation of the father

·       Unemployed

·       Housewife/Housemaid

·       Unskilled

·       skilled

·       Shop owner

·       Clerical

·       Semi-professional

·       Farmer

·       Professional

13. Occupation of the mother

Unemployed

Housewife/Housemaid

Unskilled

skilled

Shop owner

Clerical

Semi professional

Farmer

Professional

14. Total number of family members:

15. Monthly income of the family:

16. Socio-economic status of the family:

17. Family history of Spectacles (One or both parents)

Yes

No

Don’t know

18. Lightning at home while reading /studying

Adequate

Inadequate

19. Screen timing (TV, Mobile, Laptop, TAB) in 24 hours

Less than 1 hour

2-3 hours

4-6 hours

More than 6 hours

No screen time in 24 hours

20.Snellen chart test :

21. Ptosis in any of the eyes

Yes

No

22. Edema of the eyelid in any of the eyes

Present

Absent

23. Discharge or lesion of the eye   in any of the eyes

Present

Absent

 24. Redness in the conjunctiva of any of the eyes

Present

Absent

25. Abnormality of the pupil in any of the eyes

Present

Absent

26.  Rash around the eyes

Present

Absent

27.  Squint in any of the eyes

Present

Absent

28. Excessive blinking 

Present

Absent

29. Rubbing the eyes frequently

Present

Absent

30. Strabismus

Present

Absent

31. Reasons for non-utilization of eye health services
